# Cytoreductive Surgery and HIPEC for Regionally Advanced Gallbladder Cancer: a Case Report

**DOI:** 10.1007/s13193-022-01634-3

**Published:** 2022-08-29

**Authors:** Fay Huang, Raphael Shamavonian, David L. Morris

**Affiliations:** 1 Hepatobilliary and Surgical Oncology Unit, Department of Surgery, St George Hospital, University of New South Wales, Level 3, Clinical Sciences (Pitney) Building, Kogarah, NSW 2217 Australia; 2 St George and Sutherland Clinical School, University of New South Wales, Sydney, NSW Australia; 3 School of Medicine, University of Notre Dame, Sydney, NSW Australia

**Keywords:** Cytoreductive surgery, HIPEC, Gallbladder cancer, Survival

## Abstract

Gallbladder cancer is a rare cancer, associated with an extremely poor prognosis. Cytoreductive surgery and hyperthermic intraperitoneal chemotherapy is not commonly performed in gallbladder cancer; however, case series have shown prolonged survival time with cytoreductive surgery and hyperthermic intraperitoneal chemotherapy in gallbladder cancer and no increase in morbidity compared to cytoreductive surgery without hyperthermic intraperitoneal chemotherapy. We present a case of gallbladder cancer with peritoneal metastases in a 60-year-old male who was successfully treated with complete cytoreductive surgery and hyperthermic intraperitoneal chemotherapy and survived for 4 years following diagnosis.

## Introduction

Gallbladder cancer (GBC) is often diagnosed at an advanced stage, resulting in a poor prognosis, with a median survival of 1 year [1, 2]. Cytoreductive surgery (CRS) and hyperthermic intraperitoneal chemotherapy (HIPEC) has potential to treat peritoneal metastases; however, it is not commonly performed in GBC [3–5]. We present a case of GBC with peritoneal metastases in a 60-year-old male who was successfully treated with complete CRS and HIPEC and survived for 4 years following diagnosis.

## Case Report

**Fig. 1:**
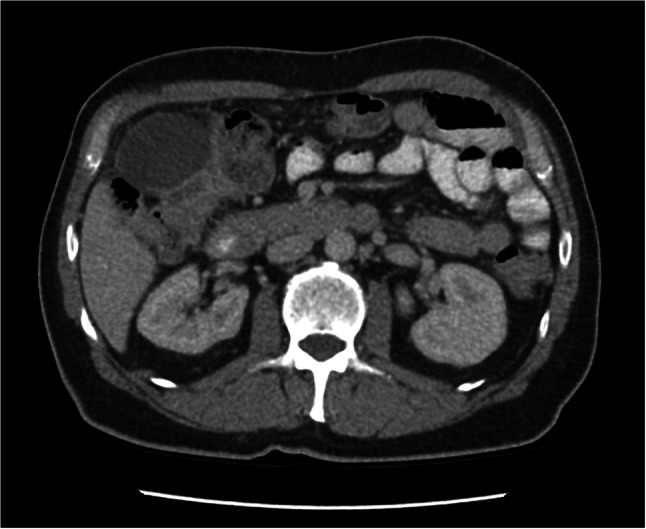
Axial view of abdominal CT showing mass at hepatic flexure

A 60-year-old male presented to a regional hospital with a 4-day history of lower abdominal pain, constipation and 10-kg unintentional weight loss. He was otherwise well with no past medical history, no regular medications and no family history of malignancy. On examination, he was haemodynamically stable and tender in the lower abdomen. A computed tomography (CT) scan showed a 4-cm obstructing sigmoid colon mass as well as a hepatic flexure mass extending to the gallbladder, stomach and duodenum (Fig. 1). On bloods, CEA was 11 ng/mL, CA 19.9 570 U/mL, CA 125 90 IU/mL, and AFP was 2 kU/mL. Other bloods including liver function tests were unremarkable.

**Fig. 2:**
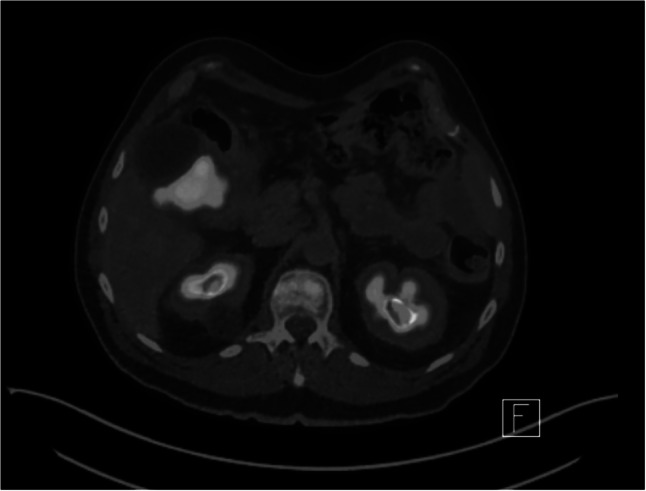
Axial view of PET showing avid tracer uptake adjacent to the hepatic flexure

**Fig. 3:**
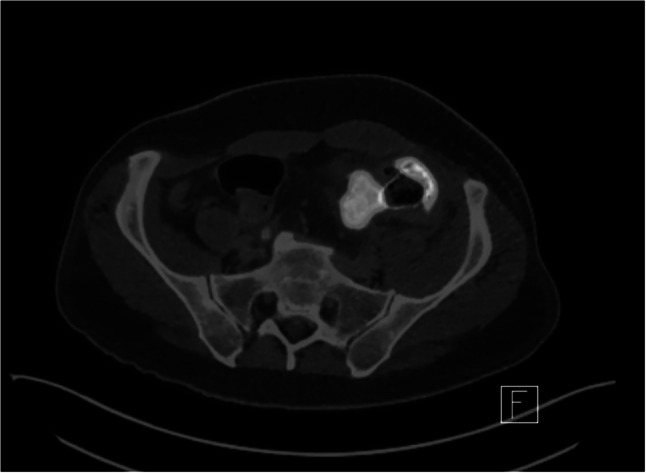
Axial view of PET showing tracer uptake at the sigmoid colon, with colonic stent in situ

The patient underwent a colonoscopy and stent insertion, at which time a gastroscopy was also done which did not demonstrate a lesion extending into the stomach. He was further investigated with positron emission tomography (PET) which showed avid tracer uptake adjacent to the hepatic flexure and at the sigmoid colon consistent with neoplasia (Figs. 2 and 3). Multiple peritoneal nodules and portal nodes were also identified on PET. There were no metastases to liver, lung, or bone.

The patient was transferred to a metropolitan hospital for further treatment where he underwent CRS and HIPEC for regionally advanced disease from assumed sigmoid colon primary. He had a peritoneal carcinomatosis index (PCI) of 14. A peritonectomy was performed which included cholecystectomy, B2 gastrectomy, right hemicolectomy, anterior resection and formation of ileostomy. Oxaliplatin was the agent used during HIPEC. The completeness of cytoreduction (CC) score following CRS was 0. The post-operative course was complicated by a pancreatic leak, pulmonary embolism and pseudomonas bacteraemia, which were all managed appropriately. The patient was discharged on day 24 following his surgery.

Histopathology revealed a primary gallbladder adenocarcinoma with multiple metastatic deposits in the small bowel, duodenum, transverse colon and transmural infiltration of the sigmoid colon. Of the 11 paracolic lymph nodes resected, 3 were positive for adenocarcinoma.

The patient completed eight cycles of adjuvant chemotherapy of gemcitabine and cisplatin. He remained disease free at 2-year follow-up. In the following year, he developed a solitary lung nodule which was treated with radiotherapy. Further metastases soon became apparent to his liver and bones. The patient died 4 years after diagnosis from aspiration pneumonia following a pathological femoral fracture.

## Discussion

Gallbladder cancer (GBC) is rare, accounting for 1.2% of all cancer diagnoses [6]. It affects 2–3 in 100,000 people globally and women 2–3 times more commonly than men [6, 7]. The main type of GBC is adenocarcinoma [6, 7]. GBC often presents with non-specific symptoms such as abdominal pain, fevers, jaundice and weight loss [8]. It is therefore often diagnosed at late stage, with up to 80% of American cases showing regional or distant metastases at presentation [2]. As a result, GBC is associated with poor prognosis and median survival less than 12 months or up to 14 months with surgical resection [1, 2, 6]. On an autopsy study, the most common sites of local infiltration by GBC included liver (64.8%), colon (15.3%) and duodenum (14.6%) [9]. Common sites of metastases were liver (66%), lungs (15%) and skeletal (9.4%) [9]. Peritoneal spread was not commonly found [9]. The 5-year survival rate for distant metastasis is 2%, with 28% for regional disease and 60% for localised disease [2].

Risk factors include chronic cholecystitis, cholelithiasis, environmental exposures, obesity, family history and elevated cholesterol which may be linked to elevated oestrogen levels [6, 7]. There is currently no standardised screening method for GBC, and tumour markers are not specific to the disease, making it difficult to diagnose in early stages [7]. The most frequently elevated markers are CA19.9 and CEA in advanced stages of GBC [7]. Standard workup includes general blood tests, especially liver function tests, full blood count and tumour markers such as CA19.9, CA125, CA242, CEA and CA15.3 [7, 8]. Imaging such as ultrasound; CT of the chest, abdomen and pelvis; magnetic resonance imaging or magnetic resonance cholangiopancreatography, as well as PET, are frequently used in staging the disease [8]. Endoscopic ultrasonography may also be beneficial in differentiating GBC and benign lesions of the gallbladder [8].

There is ongoing debate regarding optimal treatment modality of GBC. It is often treated with palliative chemotherapy, but treatment options include curative or palliative chemotherapy, radiotherapy and surgical resection [3, 10–13]. Surgical management ranges from cholecystectomy alone to complete CRS [3, 10–13]. Some studies have indicated that more aggressive resection achieves longer survival time compared to cholecystectomy alone, and we have presented a case where the survival time after CRS with HIPEC is almost 3 years longer than the median survival time for GBC [12, 14]. Adjuvant chemotherapy with cisplatin and gemcitabine, which our patient completed, has also been associated with longer survival [11].

CRS with HIPEC has demonstrated improved long-term survival for patients with various peritoneal malignancies, including colorectal cancer, appendiceal and primary pseudomyxoma peritonei [3–5, 15]. However, limited data exists regarding its use in GBC with peritoneal metastasis, with only a handful of cases described in the literature [3–5, 15]. Randle et al. presented 5 patients who underwent CRS with HIPEC for GBC with peritoneal metastases and reported a 30% 3-year survival rate and median survival time of 22.4 months after CRS [15]. The patients in this series had low volume disease; however, no specific PCI was reported. All were diagnosed with GBC from laparoscopic cholecystectomy and had a mean delay of 20 months between diagnosis and CRS, suggesting potentially less aggressive disease. They reported 17% major post-operative morbidity but no mortality, and the mean length of hospital stay was 9 days [15]. Leigh et al. presented 3 GBC patients who underwent CRS with HIPEC; however, PCI ranged from 7 to 27, and complete cytoreduction was only achieved in 1 patient [13]. Median survival time was 8 months, and all 3 patients died within 13 months, with the patient who had CC-0 surviving the longest [13]. A recent Korean centre reported another case of GBC with limited peritoneal seeding, treated curatively with CRS and HIPEC [3]. Their patient also had complications of a pancreatic leak and required 18-day stay post-operatively. Their patient underwent 6 cycles of adjuvant cisplatin, gemcitabine and abraxane and was still alive at 1 year follow up [3]. Kang et al. presented patients with locally advanced GBC who underwent curative complete CRS without HIPEC and showed an overall median survival time of 8 months [11]. Another case series compared 35 patients who underwent CRS and HIPEC to 43 who only had surgery and adjuvant chemotherapy and showed a statistically significantly longer survival time of 19 months in the HIPEC group compared to 15 months in the non-HIPEC group [16]. Although it reported a statistically significant longer hospital stay in the HIPEC group, HIPEC did not increase the rate of post-operative complications [16].

We presented a case where a patient achieved 4-year overall survival following CC-0 with CRS and HIPEC. This is substantially longer compared to the expected 14-month survival time for GBC treated with surgical resection [1, 2, 6]. Other case series using CRS and HIPEC have also shown median survival time longer than 14 months, except for Leigh et al. [13] where complete cytoreduction was obtained in only 1 patient, suggesting the PCI and extent of cytoreduction may affect survival. The median survival time was also shorter in groups which did not use HIPEC, suggesting the potential benefits of the addition of HIPEC to surgical resection [11, 16]. Importantly, it was noted that the use of HIPEC did not increase post-operative complications [16].

## Conclusion

GBC with peritoneal metastasis carries a dismal prognosis with current palliative systemic therapies. We demonstrated a case where a patient with peritoneal metastasis from GBC was able to achieve 4-year overall survival, including 2 years disease free, following CRS and HIPEC. Traditionally considered a terminal disease, select patients may achieve modest survival benefit from CRS and HIPEC in GBC with peritoneal metastasis.
